# Separating arbitrary free-space beams with an integrated photonic processor

**DOI:** 10.1038/s41377-022-00884-8

**Published:** 2022-07-05

**Authors:** Maziyar Milanizadeh, SeyedMohammad SeyedinNavadeh, Francesco Zanetto, Vittorio Grimaldi, Christian De Vita, Charalambos Klitis, Marc Sorel, Giorgio Ferrari, David A. B. Miller, Andrea Melloni, Francesco Morichetti

**Affiliations:** 1 Department of Electronics, Information and Bioengineering, Politecnico di Milano, via Ponzio 34/5, 20133 Milano, Italy; 2 School of Engineering, University of Glasgow, Glasgow, G12 8QQ UK; 3 TeCIP Institute, Scuola Superiore Sant’Anna, 56124 Pisa, Italy; 4 Ginzton Laboratory, Stanford University, Spilker Building, Stanford, CA 94305 USA

**Keywords:** Silicon photonics, Photonic devices

## Abstract

Free-space optics naturally offers multiple-channel communications and sensing exploitable in many applications. The different optical beams will, however, generally be overlapping at the receiver, and, especially with atmospheric turbulence or other scattering or aberrations, the arriving beam shapes may not even be known in advance. We show that such beams can be still separated in the optical domain, and simultaneously detected with negligible cross-talk, even if they share the same wavelength and polarization, and even with unknown arriving beam shapes. The kernel of the adaptive multibeam receiver presented in this work is a programmable integrated photonic processor that is coupled to free-space beams through a two-dimensional array of optical antennas. We demonstrate separation of beam pairs arriving from different directions, with overlapping spatial modes in the same direction, and even with mixing between the beams deliberately added in the path. With the circuit’s optical bandwidth of more than 40 nm, this approach offers an enabling technology for the evolution of FSO from single-beam to multibeam space-division multiplexed systems in a perturbed environment, which has been a game-changing transition in fiber-optic systems.

## Abstract (web summary)

We show that a programmable photonic integrated processor can separate, directly in the optical domain, spatially-overlapped free-space optical beams with unknown shapes, sharing the same wavelength and polarization.

## Graphical abstract

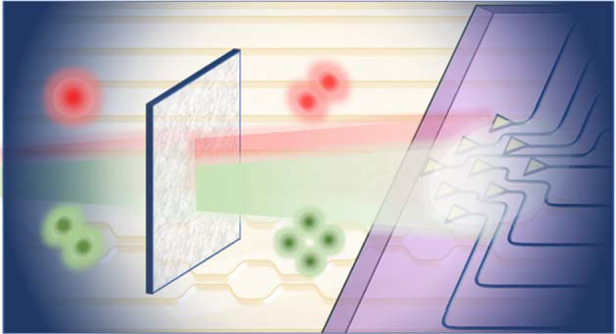

## Introduction

The concepts of space diversity and space multiplexing are well established in communications systems and are widely employed in microwave wireless systems to implement high-capacity multiple input - multiple output (MIMO) links. In the optical domain, space-division multiplexing (SDM) has been known for several decades^1^, but only recently it has started to be seriously considered as a strategy to face the capacity crunch of optical fibers^2^. Fiber optic SDM systems exploit multicore or few-mode fibers to increase the spectral efficiency (in terms of bit/Hz/s) of the transmitted signal, but the price to be paid at the receiver is the need for coherent detection assisted by electronic digital signal processing (DSP). Such a DSP should run at the bit rate to recover the signal integrity by undoing the mode mixing occurred during fiber propagation. To reduce the power consumption and the speed limitations of the DSP, several solutions have been proposed to perform all-optical demultiplexing and unmixing of optical guided modes at the receiver^3–7^.

The same evolution that fiber optic communications has experienced towards SDM systems is now happening in Free-Space Optics (FSO). FSO communication is attracting renewed and ever-increasing interest because it is a potential solution to meet the growing demand for wireless bandwidth and the low latency requirements of Internet of Things (IoT) technologies in next generation networks^8^. As in the case of fiber optic communications, SDM in FSO requires the use of orthogonal sets of beams (or modes), and several pioneering demonstrations have been achieved by using orbital angular momentum (OAM) modes^9,10^, Bessel beams^11^, and Laguerre-Gauss (LG) modes^12^. To generate a number of orthogonal beam configurations, and to demultiplex them at the receiver, the light beams have to be shaped according to suitable amplitude, phase, and polarization profiles^13^. Traditionally, these operations are performed by using bulk optics, such as classical lenses and diffractive elements. Spatial light modulators (SLMs) offer more flexibility and reconfigurability in beam manipulation^14^; however, SLMs have some limitations, such as a relatively low speed (a few hundred Hz), the possibility of modifying only the phase of light (only a few examples use amplitude control^15^, with efficiency penalties) and the need for computationally heavy calibration techniques. Also, interacting once with a single diffractive element or SLM does not allow the separation of arbitrary beams^16^. With careful design, multiple planes of SLMs or diffractive optical elements (or, equivalently, successive interactions with different regions of the same element) can separate known mode families, such as Laguerre-Gauss beams^17^, but adaptation to separating arbitrary beams is challenging, especially in real time and for unknown beams.

A powerful alternative technology for the manipulation of the FSO beams is offered by programmable photonic processors. These circuits are general-purpose photonic integrated circuits, made of meshes of tuneable integrated interferometers, that can implement arbitrary linear transformations^18^. Because of their flexibility, they have been already used in many different applications, including reconfigurable filters^19^, unmixing of guided modes^4^, vector-matrix multiplication and computing^20^, quantum information processing^21^ and neural networks^22,23^, By connecting the input/output ports of these architectures to integrated optical antennas, these processors can be used for on-chip manipulation of FSO beams. For instance, we employed a silicon photonic mesh of Mach-Zehnder interferometers (MZIs) to control the complex field radiated by a 1D array of optical antennas, demonstrating several functionalities like the generation of perfectly shaped FSO beams with nonperfect optical antennas and imaging through a diffusive medium^24^.

In this work, we show that a programmable photonic processor can separate, directly in the optical domain, overlapped arbitrary FSO beams that are orthogonal within a certain basis, even when they share the same wavelength and polarization. The circuit implements an adaptive multibeam receiver for FSO systems, which can recover the information carried by the received spatially-overlapped FSO beams with negligible mutual crosstalk. Several examples are provided including pairs of beams arriving from orthogonal directions (*direction-diversity*), as well as beams arriving from the same direction but shaped according to different orthogonal spatial modes (*mode-diversity*), and even when the two orthogonal beams have undergone some mixing during propagation.

## Results

### Multibeam free-space optics receiver

**Fig. 1:**
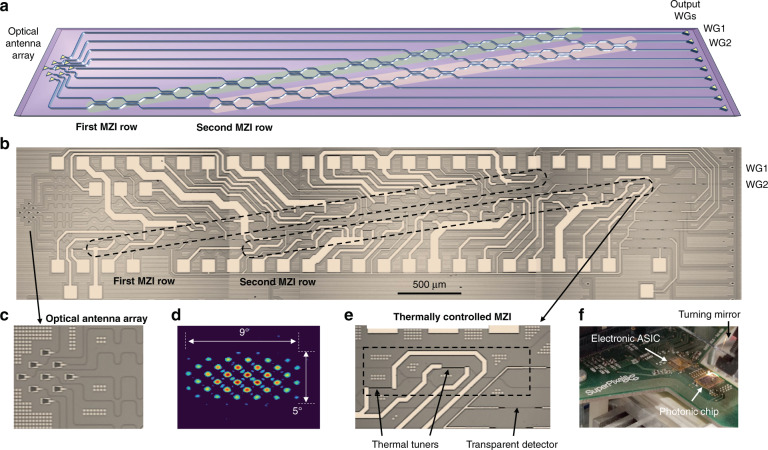
Multibeam FSO Receiver. **a** Schematic of a 9 × 2 diagonal photonic processor comprising two rows of tunable MZIs and implementing a two-beam FSO receiver. The 2D optical antenna array is used to couple free-space beams into the silicon waveguides, while the output ports WG1 and WG2 are used to couple the light out to a pair of optical fibers. **b** Microscopic picture of the fabricated silicon chip. **c** Detail of the 2D optical antenna array made of 3 × 3 grating couplers in a square configuration. **d** Measured far-field pattern radiated by the 2D optical antenna array when all the grating couplers are excited with the same amplitude and phase. Multiple diffraction orders (grating lobes) are visible within the 5° × 9° angular beamwidth of the radiation pattern of the elementary grating coupler. **e** Detail of a thermally tunable beam coupler with a transparent monitor detector integrated at output ports. **f** Photograph of the photonic chip assembled on a PCB integrating the CMOS electronic ASIC for the read-out of on-chip detectors

The integrated photonic processor employed in this work consists of a mesh of tunable beam splitters, which are realized by means of balanced MZIs. The topology of the circuit is shown in the schematic of Fig. 1(a), which includes *N* = 2 rows of cascaded MZIs^25^. On the left side of the mesh, a 2D array of *M* optical antennas (*M* = 9 in this particular device) is employed as an input/output interface between FSO beams and the guided modes of an array of single-mode optical waveguides. The optical antennas are implemented by using standard grating couplers typically used to couple the light with optical fibers; however, the presented results can be extended to integrated photonic processors terminated with arbitrary optical antennas, whose individual radiation diagram can be optimized for specific applications. On the right side of the mesh, two of the output waveguides, WG*n* (*n* = 1, 2), are used as output ports; the remaining 7 waveguide outputs are available for monitoring and control purposes.

Such a processor can be used to separate, essentially losslessly, any two “orthogonal” input beams to these two output ports, and this can be accomplished by a progressive self-configuring algorithm based on single-parameter power minimization feedback loops, without calculations. Formally, by “orthogonality” of two beams here we mean two beams that lead to orthogonal complex vectors of amplitudes in the *M* input waveguides inside the processor. To the extent that two different (and possibly overlapping) input beams lead to such orthogonal vectors, this processor can separate them essentially losslessly and automatically. More generally, a processor with *M* input ports and *N* output ports can separate up to *N* arbitrary beams belonging to a set of *M* orthogonal beams. The mathematics of these kinds of mesh processors and their operation as self-configuring systems is well understood and has been described extensively^24–27^. For completeness and clarity, we also describe the basic mathematics and operation of this particular processor in the Supplementary Material (see Section S1).

This ability to separate such “orthogonal” beams is here exploited to implement a multibeam receiver for FSO beams. A light beam shining on the chip is sampled by the 2D array of *M* optical antennas and is coupled to the single-mode waveguides at the input of the programmable photonic processor. The light field in these *M* waveguides can be coherently summed by configuring the first row of MZIs in such a way that the light in a first beam is entirely extracted out of WG1 with no residual power transmitted at the other output ports^25^. One can then simultaneously shine a second free-space optical beam, orthogonal to the first one (in the sense of having an orthogonal vector of amplitudes at the input optical antennas) onto the 2D antenna array; this second beam can share the same wavelength and polarization as the first beam. As discussed in detail in the following sections, this kind of orthogonality between the two beams can result from a different arrival direction or a different spatial shape, typically referred to as mode orthogonality. When the second beam is shone onto the photonic chip, its field is spatially sampled by the 2D antenna array and co-propagates with the first beam in the same *M* single-mode waveguides, thus being apparently indistinguishable inside each waveguide. However, because of the orthogonality, no portion of the second beam is transmitted to output port WG1. The second row of MZIs stages can be used to coherently reconstruct the power of the second beam in the output port WG2. Generalizing the concept to a photonic processor with *M* inputs and *N* rows of MZIs (with *M* > *N*), we can conclude that such a device can couple, reconstruct and separate *N* orthogonal free-space beams (actually *N* + 1 if *N* = *M*-1), which are spatially sampled by *M* optical antennas, and transmit them to *N* single mode output waveguides, with arbitrary sorting order and no mutual optical crosstalk. This is the basic concept of the multibeam FSO receiver developed in this work.

If the propagation of the light is reversed, the photonic processor can operate as a multibeam transmitter^26^, enabling us to map the light intensity carried by *N* single mode waveguides into *N* orthogonal free-space beams. By tuning the integrated MZIs, both the amplitude and the phase of the light radiated by each element of the 2D optical antenna array can be controlled. In this way the shape and the direction of the far-field beam can be modified. Note that these approaches change amplitudes by re-routing the light, not by absorbing or otherwise attenuating the beam, so there is no fundamental loss as relative amplitudes are adjusted. The number *M* and the positions of the optical antennas set the spatial basis set^27^ for the generation and collection of the free-space beams, and *M* sets the number of degrees of freedom that we have for beam manipulation^24^. Even though in this work we restrict the analysis to the use of the photonic processor as a multibeam receiver, backward propagation is exploited to understand better the behavior of the device under the conditions that optimize its performance.

Figure 1(b) shows a top-view microscopic picture of the entire chip, which has a footprint of 5.8 mm × 1.3 mm. As shown in the detail of Fig. 1(c), the 2D optical antenna array is made of *M* = 9 identical grating couplers, which are all aligned in the same direction and are arranged in a 3×3 square configuration. The center-to-center spacing between the grating couplers of the 2D optical antenna array is 49 μm (corresponding to about 32λ), which leads to the presence of several diffraction orders (grating lobes) in the far-field radiation pattern with a minimum angular spacing of about 1.7°. As an example, Fig. 1(d) shows the collimated far-field intensity profile measured with a near-IR camera for a uniformly excited array (i.e., when all the elements radiate a light beam with same intensity and phase). All the 15 MZIs of the mesh (8 MZIs in the first row, 7 MZIs in the second row) are identical and are controlled by means of thermal tuners [see Fig. 1(e)]. Transparent photodetectors^28^ are used to locally monitor the switching state of each MZI to implement automatic tuning and stabilization procedures. The photonic chip was mounted on an electronic printed circuit board (PCB) [see Fig. 1(f)], housing the electronic ASIC for the read-out of the on-chip detectors^29^ and the connections to a FPGA-based board for real-time data processing and the actuation of the thermal tuners. More details on chip design and fabrication are provided in the Materials and Methods section. Unless otherwise specified, all the experiments reported in the following sections are performed at a wavelength of 1550 nm.

### Direction-diversity receiver

**Fig. 2:**
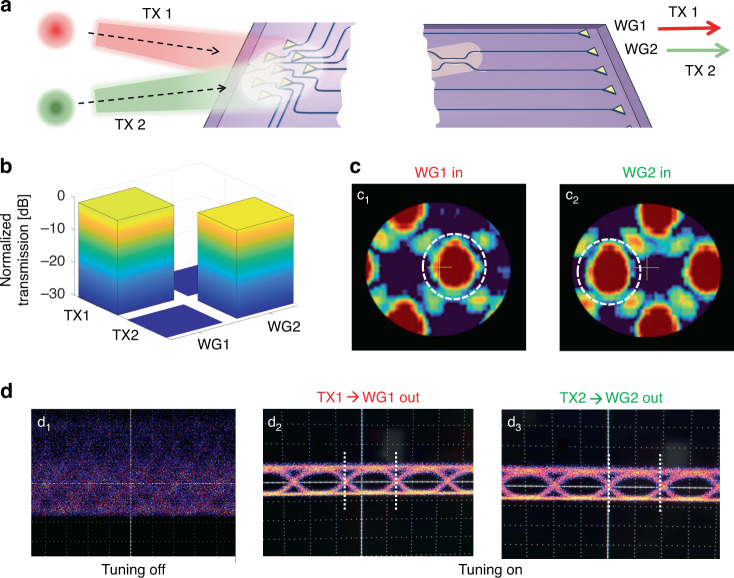
Direction-diversity receiver. **a** Schematic representation of two free-space beams (TX1 and TX2), sharing the same wavelength and state of polarization, and arriving at the receiver from different directions. **b** Bar chart showing the normalized insertion loss of the beams TX1 and TX2 (relative angle 1.25°) at the output waveguides WG1 and WG2. **c** Backward far-field intensity pattern radiated by the 2D optical antenna array when the photonic processor is configured to couple beam TX1,2 to WG1,2 launching the light from WG1 (c_1_) and WG2 (c_2_). **d** Measured eye diagrams of two received intensity modulated 10 Gbit/s OOK signals transmitted by using the two beams TX1 and TX2: (d_1_) when the photonic processor is not configured, the eye diagrams of TX1 and TX2 at port WG1 are severely overlapped; while after the configuration of the photonic processor both eyes, TX1 at port WG1 (d_2_) and TX2 at port WG2 (d_3_) are clearly open with neither evident distortion nor inter-symbol interference

As a first example of an application, we show that the integrated photonic processor can operate as a *direction-diversity* receiver, that is, a multibeam receiver capable of individually detecting beams that simultaneously arrive from different directions. This concept is demonstrated here by considering the case of *N* = 2 beams, but this functionality can be generalized to *N* beams arriving from *N* directions utilizing a photonic processor with *N* rows of MZIs. As shown in the scheme of Fig. 2(a), two free-space beams with identical Gaussian shape, wavelength (1550 nm) and polarization status (TE polarization, to match the polarization sensitivity of the grating couplers) are shone from two different directions onto the 2D optical antenna array of the photonic processor. This implies that, in this case, orthogonality is given only by the direction of arrival of the beams, which is obtained by different positions of the two transmitters, conveniently labeled as TX1 and TX2. Orthogonality conditions for the direction diversity receiver are discussed in the Supplementary Sec. S2. The aim of this experiment is to demonstrate that the photonic processor can effectively separate the two beams TX1 and TX2 at the two output ports WG1 and WG2 (or vice versa) with a negligible residual crosstalk. Details on the experimental setup employed to generate the two beams TX1 and TX2 and to test the direction-diversity receiver are given in the Supplementary Sec. S3.1 and Fig. S4.

The beam impinging on the 2D optical antenna array from each source can be individually coupled to the desired output waveguide (WG1 or WG2) of the photonic processor through automatic tuning and stabilization algorithms applied to each row of the photonic processor^4,30^, (see “Materials and Methods” section). Upon tuning the rows of the photonic processor to extract each transmitted beam, identical optical powers from two collimators are measured at ports WG1 and WG2, which is reported as 0 dB normalized insertion loss in bar chart of Fig. 2(b). The end-to-end loss from each collimator to output waveguide WG1 or WG2 is about 28 dB, accounting for the on-chip losses, the coupling loss of the grating couplers, the geometric loss of the 2D array, and the loss of the free-space optics (see “Materials and Methods” section for the loss break-down). In this experiment, the two beams arrive overlapped at the inputs of the photonic processor from different directions with a relative angle of 1.25°. Notably, more than 25 dB optical crosstalk suppression, meaning TX1 in WG2 and TX2 in WG1, is measured and reported in the same bar chart. If we swap the mode sorting status, meaning coupling TX2 to WG1 and TX1 to WG2, the same level of optical isolation is observed.

To better understand the behavior of the photonic processor in the tuned state, we reversed the direction of the light propagation by injecting the light into WG1 and WG2 ports and measuring the far-field pattern radiated by the 2D optical antenna array when the photonic processor is configured in the case of Fig. 2(b). For clarity, we restrict the view to one period of the radiation pattern, which is zoomed in around the zero-order diffraction (main lobe). Panels 2(c_1_) and (c_2_) refer to the far field radiated by the 2D optical antenna array when the light is injected from WG1 (transmission back to TX1) and from WG2 (transmission back to TX2), respectively. It can be appreciated that in each case the position of the main lobe (highlighted by the dashed circle and indicating the direction of maximum radiation) coincides with the position of the null in the other case, which is consistent with the high rejection between the two beams shown in Fig. 2(b). Notably, the high optical crosstalk suppression (> 25 dB) of the beam arriving from a different direction (1.25°) is achieved because the photonic processor can control both the amplitude and the phase of the field coupled by each grating coupler of the 2D optical antenna array (see Supplementary Section S2, Fig. S3)^24^.

Such a low crosstalk level enables us to use the integrated photonic processor as a direction-diversity receiver in a FSO communication system, where the optical beams are employed to transmit two independent data channels. To this end, the two beams TX1 and TX2 were used as carrier wavelengths for the transmission of independently modulated 10 Gbit/s on-off keying (OOK) signals. The results of the transmission experiment are shown in Fig. 2(d). When the photonic processor is not set up in any particular way [panel (d_1_)], that is when the thermal tuners of the MZIs are at arbitrary working points, the two data channels are randomly overlapped at output port WG1 and the measured eye diagram is completely closed. A similar result is observed at output port WG2 (not shown). In contrast, open eye diagrams are recorded when the photonic processor is tuned to simultaneously extract the signal TX1 at output port WG1 (d_2_) and the signal TX2 at output port WG2 (d_3_); both eye diagrams then show no degradation with respect to the reference eye diagram of the individual channels.

### Mode-diversity receiver

**Fig. 3:**
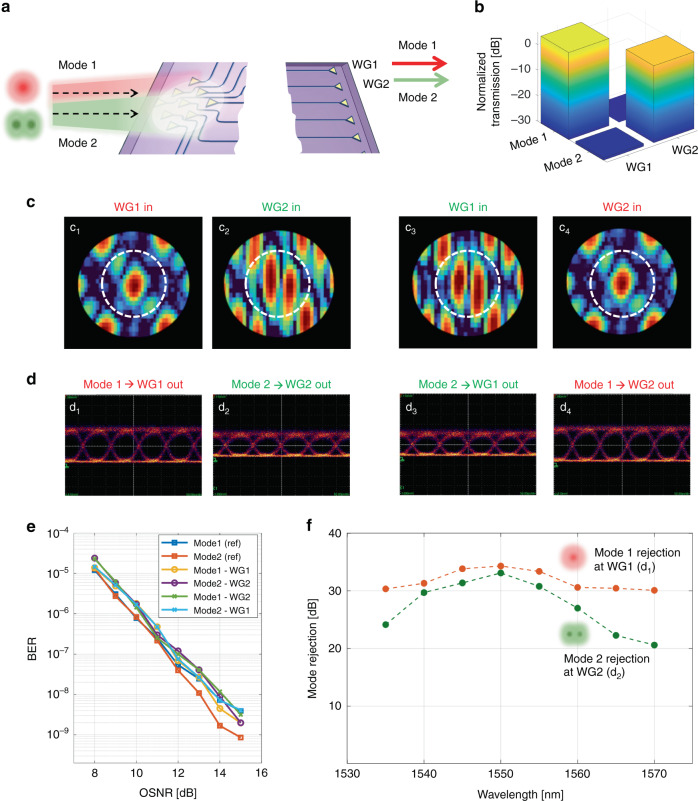
Mode-diversity receiver. **a** Schematic representation of two free-space modes (Mode 1 and Mode 2), sharing the same wavelength and state of polarization, and arriving at the receiver from the same direction. **b** Bar chart showing the normalized received power of the Mode 1 and Mode 2 at the output waveguides WG1 and WG2. **c** Backward far-field intensity pattern radiated by the 2D optical antenna array when the photonic processor is configured to couple Mode 1 to WG1 (c_1_), Mode 2 to WG2 (c_2_), Mode 2 to WG1 (c_3_) and Mode 1 to WG2 (c_4_) entering from WG1 and WG2. Circles indicate the position of the zero-order diffraction. **d** Measured eye diagrams of two intensity modulated 10 Gbit/s OOK signals transmitted by using Mode 1 and Mode 2 for the configurations considered in (**c**). **e** BER measurements of 10 Gbit/s OOK channels simultaneously transmitted in free space on spatially overlapped modes (Mode 1 and Mode 2) and separated by the photonic processor. Blue and red squares indicate the reference BER measured when only one data channel is switched on. Negligible OSNR penalty is observed for any data channels sorted out by the photonic processor. **f** Wavelength dependence of the mutual mode rejection at the output ports of the photonic processor

In principle, the coupling, separation, and sorting of free-space beams can be achieved by the programmable photonic processor on any set of orthogonal beams. As a second example, we consider two free-space beams, sharing the same wavelength (1550 nm) and state of polarization (TE), and coming from the same direction, yet being shaped according to different orthogonal spatial modes. In this case, the programable photonic processor receiving these two beams operates as a multibeam *mode-diversity* receiver. This situation is schematically shown in Fig. 3(a), where two free-space beams, labeled as Mode 1 and Mode 2, simultaneously impinge on the 2D optical antenna array; the photonic processor is configured to separate them at the two output ports WG1 and WG2 (or vice versa). In the experiment described in the following, Mode 1 and Mode 2 refer to the fundamental Hermite-Gaussian mode HG00 and a higher order HG10-like mode, respectively (for details on the experimental setup see Supplementary Section S3.2, Fig. S5).

Adopting the same automated procedure described before for configuration of the circuit (see Materials and Methods section), the first MZI row of the photonic processor is lined up to maximize the coupling from one of the two modes (for instance Mode 1) to the output port WG1, which leads to nulling of the other mode (Mode 2) at this port. The bar chart of Fig. 3(b) shows the relative transmission of the two modes measured at both output ports after the configuration of the photonic processor. An intensity ratio of more than 30 dB between the extracted mode and the rejected mode is observed at both ports. If mode sorting is swapped, that is if Mode 2 is coupled to WG1 and Mode 1 is coupled to WG2, the same level of isolation (more than 30 dB) is obtained.

By reversing the propagation direction of the light, the far-field profile of the beam generated by the 2D optical antenna array can be observed for the different configurations of the photonic processor. All the possible cases handled by the two-diagonal photonic processor are reported in Fig. 3(c). For instance, let us consider the situation where the first row of MZIs is configured in forward propagation to couple Mode 1 to output port WG1; in the reversed direction—that is, when WG1 is used as an input port – the far field radiated back by the 2D optical antenna array is well shaped as the fundamental HG00 mode [panel (c_1_)]. If the second MZI row is configured to couple Mode 2 to WG2, the far field radiated back when WG2 is used as an input port is shaped like the HG10-like mode [panel (c_2_)]. Panels c_3_ and c_4_ show the far-field pattern for the opposite coupling scenario. Notably, in all these cases the photonic processor automatically self-configures by simply minimizing the power of the relevant mode at each stage of the MZI rows (see “Materials and Methods” section), without any prior knowledge of the incoming beam shapes.

The performance of programmable photonic processor as a multibeam mode-diversity receiver was assessed by means of data channel transmission. Two intensity-modulated 10 Gbit/s OOK data streams were transmitted on the spatial and direction overlapped modes (HG00 and HG10-like) at the same carrier wavelength of 1550 nm and the same polarization state. The eye diagrams of the received signals, after the separation performed by the photonic processor, are shown in Fig. 3(d) for all the configurations shown in Fig. 3(c). No degradation due to the residual mutual optical crosstalk from the interfering orthogonal mode can be observed. As a quantitative assessment of the effectiveness of the mode separation performed by the photonic processor, the bit error rate (BER) of the received channels was measured versus the optical signal to noise ratio (OSNR). The noise power in the OSNR is evaluated across a bandwidth equal to the bandwidth of the signal. Figure 3(e) shows the BER curves measured on the received data channels encoded onto the modes HG00 (Mode1) and HG10-like (Mode2). As reference curves, we measured the BER of the two modes (HG00 blue squares, HG10 red squares) when they are individually transmitted through the photonic processor to output port WG1 in the absence of the other mode. The other curves show the BER measured when both data channels are switched on and the modes are sorted out at the output ports WG1 and WG2 in all the four possible configurations. Thanks to the high optical crosstalk rejection between the separated modes, no significant OSNR penalty is observed in all the considered cases.

We also evaluated the wavelength range across which the photonic processor can guarantee a high isolation in the separation of the two modes. To this end, the carrier wavelength of the two modes was swept across a 35-nm-wide range from 1535 nm to 1570 nm. The width of this range is mainly limited by the wavelength-selective response of the grating couplers of the 2D optical antenna array. In the results reported in Fig. 3(f), for every wavelength considered, the photonic processor was configured to extract Mode 2 at output port WG1 and Mode 1 at output port WG2; these are the cases considered in the eye diagrams of panels (d_3_) and (d_4_) for the central wavelength of 1550 nm. The red curve shows that the intensity rejection of Mode 1 at port WG1 is higher than 30 dB across the entire wavelength range. The drop of mode rejection versus wavelength is due to the wavelength dependence of the 3-dB directional couplers of the MZIs of the photonic processor, which can be reduced by replacing the directional couplers with broad-band 3-dB multimode interference couplers^31^. The rejection is somewhat lower for Mode 2 at port WG2, yet is still higher than 20 dB across 35 nm (see Supplementary Sec. S5 for more details on the wavelength dependence of the device).

### Mode-mixed receiver

In this section, we extend the concept of the mode-diversity receiver presented in previous section to other examples of spatially overlapped beam pairs that can be disentangled by the programmable photonic processor. In particular, we show that the photonic processor can still separate two orthogonal free-space beams even after they have propagated spatially overlapped through a mode mixing obstacle or a free-space path perturbation. Mathematically, this means that the linear transformation performed by the mode mixer, which maps the original orthogonal modes to another pair of orthogonal modes, can be inverted by the photonic processor.

**Fig. 4:**
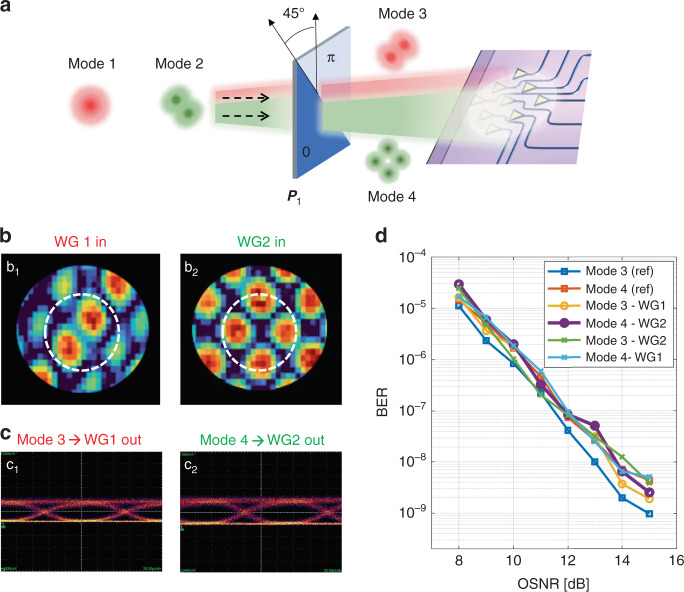
Mode separation after a controllable mode-mixing. **a** Schematic representation of two free-space modes (Mode 3 and Mode 4) that arrive on the receiver after a mode conversion performed by a 45°-rotated phase mask. In the reported experiment, Mode 3 and Mode 4 correspond to 45°-rotated HG10-like and HG11-like modes, respectively. **b** Backward far-field intensity pattern radiated by the 2D optical antenna array when the photonic processor is configured to couple Mode 3 to WG1 (b_1_) and Mode 4 to WG2 (b_2_). Circles indicate the position of the zero-order diffraction. **c** Measured eye diagrams of two intensity modulated 10 Gbit/s OOK signals simultaneously transmitted by using Mode 3 and Mode 4 for the configurations considered in (**b**). **d** BER measurements of 10 Gbit/s OOK channels simultaneously transmitted in the free space on spatially overlapped modes (Mode 3 and Mode 4) and separated by the photonic processor. Blue and red squares indicate the reference BER of individually transmitted channels

As a first example we consider the mapping of an input pair of mutually orthogonal modes to an output pair of orthogonal modes belonging to the same mode set. In this case, the process can be considered simply as a mode conversion. As shown in the schematic of Fig. 4(a), a 0-π phase mask converts the fundamental mode HG00 into a 45° rotated HG10-like mode (Mode 3), while the higher-order mode HG10-like mode (-45° rotated) is transformed to a 45° rotated HG11-like (Mode 4) (see Supplementary Section S3.3, Fig. S6). From the point of view of the photonic processor, the operation needed to be performed to separate these new modes at the output ports WG1 and WG2 is conceptually identical to the one discussed in the previous sections. In fact, no a-priori information on the shape of the incoming beams is required and the self-configuring procedure to line up the MZI rows is exactly the same. Figure 4(b)–(d) show the results that are achieved when the photonic processor is tuned to extract Mode 3 at output port WG1 and Mode 4 at output port WG2. From the intensity ratio of the extracted modes at each output port, we observe more than 30 dB mutual isolation. Figure 4(b_1_) and (b_2_) show the NIR camera acquisition of the far-field patterns that are radiated back by the photonic processor when the light is injected from port WG1 and WG2, respectively. A very good matching with the shape of the HG10-like mode and of the HG11-like mode (both rotated by 45°) is found. The good mode separation is confirmed when the photonic processor is used as a two-beam mode-diversity receiver in a data transmission link, where each mode carries a 10 Gbit/s intensity-modulated OOK signal. Neither distortion nor inter-symbol interference effects are visible in the received eye diagrams of Fig. 4(c_1_)-(c_2_), which refer respectively to the data channel transmitted on Mode 3 extracted at output port WG1 and to the data channel transmitted on Mode 4 extracted at output port WG2. Figure 4(d) shows the BER curves versus OSNR measured on the data channels separated by the mode-diversity receiver. With respect to the reference curves, given by the BER curves of Mode 3 (blue squares) and Mode 4 (red squares) when they are individually transmitted in the absence of the other mode, no OSNR penalty is observed when both data channels are transmitted and they are sorted out by the photonic processor at the output ports WG1 and WG2. These results validate the effectiveness of the photonic processor to separate generic pairs of orthogonal modes emerging from a free-space mode converter.

**Fig. 5:**
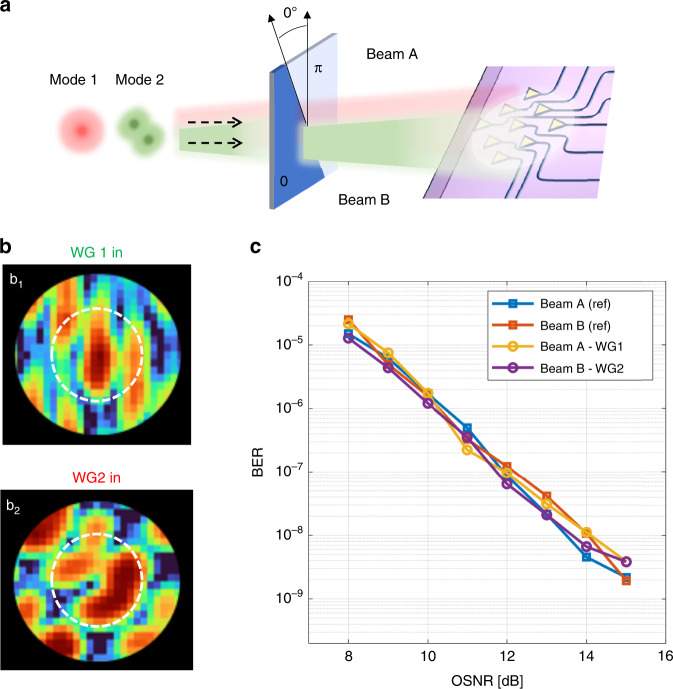
Mode separation after an arbitrary mode-mixing. **a** Schematic representation of two free space beams (Beam A and Beam B) that arrive on the receiver after an unknown linear transformation performed by a randomly oriented phase mask acting on two orthogonal modes (Mode 1 is HG00 and Mode 2 is 45° rotated HG10-like). **b** The shape of Beam A and Beam B can be identified by observing the backward far-field intensity pattern radiated by the 2D optical antenna array when the photonic processor is configured to couple Beam A to WG1 (b_1_) and Beam B to WG2 (b_2_). Circles indicate the position of the zero-order diffraction. **c** BER measurements of 10 Gbit/s OOK channels simultaneously transmitted on the overlapped Beams A and B and separated by the photonic processor. Blue and red squares indicate the reference BER of individually transmitted channels

As a final example, we shown that the photonic processor can separate beams that have experienced a generalized mode conversion process (mode mixing) in which the input modes are mapped to output modes that belong to a different mode set and do not belong to any mode family. To prove this concept, let us consider the case of Fig. 5(a) where the 0-π phase mask responsible for the mode mixing is rotated at an arbitrary angle. Presuming that the phase mask does not introduce any relevant loss, it produces an arbitrary mixing of the incoming modes, resulting in the generation of two beams, namely Beam A and Beam B, that are still orthogonal, but which no longer resemble any of the modes of the HG family (though they are still describable as some linear combination of HG-like modes, with possibly more than two terms). The shape of these beams is not known if the axis of the phase mask is unknown. Nonetheless, the configuration of the photonic processor to separate them can be operated as discussed in the previous examples without any a-priori information on the incoming beams. If we want to know the shape of the two Beams A and B, we can reverse the direction of propagation, injecting the light at ports WG1 and WG2 and looking at the far field radiated by the 2D optical antenna array with the NIR camera. The field profiles of Beam A and Beam B are shown in Fig. 5(b_1_) and 5(b_2_) and, as expected, they exhibit an arbitrary shape that does not match any of the tabulated optical free-space modes. Nonetheless, they are still orthogonal and they can be separated with extremely low mutual crosstalk, as confirmed by the BER measurements shown in Fig. 5(c).

## Discussion

In summary, we demonstrated that an integrated photonic processor can be used to separate arbitrary overlapped orthogonal FSO beams sharing the same wavelength and polarization. In the results presented, the photonic processor is operated as either a *direction-diversity* receiver or a *mode-diversity* receiver that simultaneously detects pairs of orthogonal beams even after they have undergone mode mixing. In all the cases considered, the beams are separated and arbitrarily sorted out at the output ports with negligible mutual crosstalk (>25 dB). Such beams are consistent with the general description of communications modes or mode-converter basis sets for arbitrary optical systems^27^; they do not need to correspond to any standard families, and do not need to be the same at transmitter and receiver, yet they still can have the key orthogonality properties of modes that make them independent communication channels.

For the *direction-diversity* receiver, the limit to the capability of separating overlapped beams is related to the orthogonality of the complex fields sampled by the 2D array feeding the photonic processor (see Supplementary Section S2). To increase the number of orthogonal directions (that is the number of orthogonal beams that can be separated), the number *M* of antennas of the array has to be increased in order to map the continuous space of the incoming beams into a set of complex vectors with a higher number of elements *M*. A processor with *M* input and *N* outputs can separate up to *N* arbitrary beams belonging to a set of *M* orthogonal beams. This condition applies to beams that are orthogonal in a broad sense (not only because of a different direction of arrival). Diffraction orders identify specific directions from which beams that are orthogonal in the continuum space lose their orthogonality because of the spatial sampling. As is well known from the theory of Phased Array Antennas^32^, diffraction orders can be pushed to wider angles by reducing the spacing between antennas and disappear when the spacing is less than half wavelength; the field of view of system, that is the angular range within which free-space beams can be received and separated, is ultimately limited by the radiation diagram of the elementary antenna of the array. In this work, grating couplers with a standard design are used, which have a radiation diagram with angular beamwidth of 5° × 9° (see Fig. 1(d)), but several solutions to implement broad field-of-view optical antennas have been recently proposed^33^.

Regarding the *mode-diversity* receiver, conceptually nothing changes if a strong mode mixing is introduced by an arbitrary scattering medium, which could be also time varying as in the case of atmospheric turbulence. Assuming that the scattering introduces the same loss for all beams, which would preserve unitarity (and hence orthogonality), a number N_in_ of mutually orthogonal input modes are mapped to N_out_ mutually orthogonal output modes that belong to a different mode set. If we want to describe these output modes as a superposition of known modes (e.g., HG, LG, OAM, …), we may generally need to use a large number of these modes, but there is nonetheless a set of orthogonal output functions with N_out_ = N_in_. The main advantage of our system is that, given the spatial sampling provided by the 2D array, on this discrete space, the processor automatically looks for the mode set that better describes the shape of the incoming beams. In other words, it finds the “best” orthogonal basis set with the lowest possible dimensionality. If the complex amplitude of the fields sampled by the 2D array are orthogonal in this discrete space, the beams can be separated whatever is the nature of the scattering medium responsible for the mode mixing. The capability of the receiver to separate arbitrarily mixed beams is only limited by the orthogonality of the discrete vectors that are sampled by the antenna array (see Supplementary Sec. S1).

The optical bandwidth of the photonic processor, spanning the extended telecommunication C-band (1530 nm – 1570 nm) allows its use as a receiver in high data-rate systems as well as in wavelength-division multiplexing (WDM) communication links. The photonic processor can self-configure through simple automated control strategies without the need for global multivariable optimization techniques; this property enables scalability of the proposed architecture to a larger number of optical antennas, as well as to photonic processors with a larger number of rows, which can handle more orthogonal beams simultaneously, without increasing the control complexity in proportion.

Besides the massive increase of data capacity offered by multibeam SDM transmission, the adaptive nature of the photonic processor would also allow the possibility of compensating for dynamic changes in the FSO link, caused by, for instance, moving obstacles or atmospheric turbulence, so as to establish and maintain, in real time, the optimum communication link. Finally, many applications can be envisioned that require advanced processing of FSO beams, including, for instance, wave-front sensing, phase-front mapping and reconstruction, multiple-beam transmission and imaging through scattering media, and chip-to-chip optical wireless communications.

## Materials and methods

### Chip design and fabrication

The integrated photonic processor was designed for operation around the 1550 nm wavelength range and was fabricated on a standard 220-nm SiP platform (AMF foundry). All the waveguides of the circuit are single-mode channel waveguides with a width of 500 nm. The grating couplers are designed to operate on transverse-electric (TE) polarized light. The emission angle with respect to the normal to the chip surface is 12°, while the radiation diagram has an angular beamwidth of 5° × 9°^24^. The nine waveguides connecting the grating couplers to the photonic processor share the same optical length in order to minimize the wavelength dependence of the multipath interferometer implemented by the mesh so the circuit can have the widest possible wavelength range of operation. Each MZI has two 3-dB directional couplers which are implemented by two 40-μm-long waveguides spaced by 300 nm. Two thermal tuners made of TiN metal strips (2 μm × 80 μm) are integrated in each MZI stage, one in the lower input waveguide, another in the waveguide of the upper internal interferometer arm; these enable the control of the relative phase shift between the optical fields at the input ports of the MZI, and the amplitude split ratio of the MZI, respectively. A turning mirror is positioned on top of the chip to steer the vertically emitted beam by the 2D array of grating couplers to the horizontal direction to facilitate the coupling with the free-space optical setup employed in the experiments presented in the following sections.

### Loss analysis

Here we provide the loss breakdown along the entire optical system: each grating coupler has about 4.5 dB coupling loss when coupled with standard optical fibers, which translates to about 11 dB insertion loss for a fiber-waveguide-fiber coupling (on chip loss of the photonic processor is less than 2 dB). When the free-space beam generated by the fiber collimator (TX1 or TX2) is coupled to the 2D array of the photonic processor, a geometrical loss of about 12 dB is added (see Supplementary Section 4). This loss can be effectively reduced by improving the fill factor using an array of lenslets or a photonic lantern device. Because of the reciprocity, the same loss is observed if the direction of propagation is reversed (that is the coupling of the far field generated by the 2D array with the fiber collimator), which is due to the fact that the fiber collimator couples with one diffraction order. The 50:50 beam splitter at the branching section of the experimental setup (P_3_ in Fig. S4–S6) introduces about 3 dB loss at each output port. An additional 2 dB loss is due to the aberration of optical system and possible minor alignment tolerances in the setup. Therefore, the end-to-end loss (from fiber collimator to fiber coupling with output waveguide WG1 or WG2) is about 28 dB.

### Control of the photonic processor

The control scheme for the automated self-configuration of MZI-based photonic processors though the implementation of local feedback loops was demonstrated in a previous work^30^. The photonic processor self-configures and self-stabilizes by exploiting dithering signals for the thermal tuners and thermal crosstalk mitigation strategies^34,35^. The control circuitry is a custom electronics board designed implementing two parallel electrical control chains for the calibration of the two MZI rows of the photonic processor. Each MZI is independently controlled with a local feedback loop that minimizes the optical power at the integrated monitor detectors [see Fig. 1(e)]. The integrated detectors are read using a custom designed ASIC that is wire bonded directly to the PIC to reduce the noise figure of the measurements. Dithering signals are used to identify the magnitude of deviation from the optimum bias point of the MZI tuneable couplers; to this end, different pairs of orthogonal frequencies ranging from 6 kHz to 21 kHz are employed for each MZI. The optical power measured by the detector is demodulated using the dithering frequencies to understand the tuning status of the individual MZIs and the DC currents fed to the thermal actuators are modified to minimize the evaluated error. To distinguish the two optical beams coupled from free space and co-propagating inside the waveguides of the photonic processor, each FSO beam is labeled with a suitable tone superimposed as a shallow amplitude modulation at a specific frequency that can be identified by the integrated detectors^4^. In the experiment reported in this work we used 500 Hz and 900 Hz, respectively, for the pairs of FSO beams used. The reconfiguration time of each MZI from random initial status is about 5 milliseconds, while having sub-millisecond tracking time

## Supplementary information

Supplementary Information

## Data Availability

Data underlying the results presented in this paper are available from the authors upon reasonable request.
