# Supplementary material for: Separating arbitrary free-space beams with an integrated photonic processor
Source: Light Sci Appl. 2022 Jul 5;11:197. doi: 10.1038/s41377-022-00884-8 (PMC9253306; doi:10.1038/s41377-022-00884-8)
Supplement: Supplementary file 1 — Supplementary Information [file 41377_2022_884_MOESM1_ESM.docx]

Supplementary Information

Separating arbitrary free-space beams with an integrated photonic processor

Maziyar Milanizadeh^1†^, SeyedMohammad SeyedinNavadeh^1†^, Francesco Zanetto^1^, Vittorio Grimaldi^1^, Christian De Vita^1^, Charalambos Klitis^2^, Marc Sorel^2,3^, Giorgio Ferrari^1^, David A.B. Miller^4^, Andrea Melloni^1^, Francesco Morichetti^1*^

^1^Department of Electronics, Information and Bioengineering, Politecnico di Milano, via Ponzio 34/5, 20133, Milano, Italy

^2^School of Engineering, University of Glasgow, Glasgow, G12 8QQ, UK

^3^TeCIP Institute, Scuola Superiore Sant’Anna, 56124 Pisa, Italy

^4^Ginzton Laboratory, Stanford University, Spilker Building, Stanford, CA 94305, USA

*Corresponding author: [francesco.motichetti@polimi.it](mailto:francesco.motichetti@polimi.it)

^†^These authors contributed equally to this work

**S1 Operating principle of the linear optical processor**

The light inputs in each of the optical antennas (grating couplers) on the photonic processor lead to corresponding complex optical amplitudes in the single mode optical waveguides to which they are connected. Mathematically, the complex amplitude of the field $u_{ij}$that is sampled by each optical antenna is given by the overlap integral between the field amplitude $\psi_{F,j}\left( x,y \right)$of the *j*-th FSO light beam impinging on the 2D array and the near field $\psi_{a,i}\left( x,y \right)$of the *i*-th antenna (grating coupler) of the array, that is

$u_{ij}\cong\iint{\psi_{a,i}^{*}\left( x,y \right)\psi}_{F,j}\left( x,y \right)dxdy$ (S1)

where (*x*, *y*) is the coordinate system at the entrance of the photonic processor. In eq. (S1) we consider a scalar approximation of the optical fields that holds for optical antennas emitting linearly transverse-polarized beams; a more general analysis of the mode overlap at the grating interface the can be found in [1]. We can therefore consider a column of nine-element complex vector ***u*** as the input to a 2x9 matrix *D* that describes the optical processor, with a two-element output complex vector ***v*** as the resulting output optical amplitudes in the two single-mode output waveguides WG1 and WG2 (see Fig. 1 of the main text). Explicitly, we can choose to write

$\boldsymbol{v}=D\boldsymbol{u}$ where $D=\left[ \begin{matrix} \begin{matrix} \begin{matrix} a_{1}^{*} \\ b_{1}^{*} \end{matrix} & \begin{matrix} a_{2}^{*} \\ b_{2}^{*} \end{matrix} & \begin{matrix} a_{3}^{*} \\ b_{3}^{*} \end{matrix} \end{matrix} & \begin{matrix} \begin{matrix} a_{4}^{*} \\ b_{4}^{*} \end{matrix} & \begin{matrix} a_{5}^{*} \\ b_{5}^{*} \end{matrix} & \begin{matrix} a_{6}^{*} \\ b_{6}^{*} \end{matrix} \end{matrix} & \begin{matrix} \begin{matrix} a_{7}^{*} \\ b_{7}^{*} \end{matrix} & \begin{matrix} a_{8}^{*} \\ b_{8}^{*} \end{matrix} & \begin{matrix} a_{9}^{*} \\ b_{9}^{*} \end{matrix} \end{matrix} \end{matrix} \right]$ (S2)

where the values of the elements in the vectors $\boldsymbol{a}=\left[ \begin{matrix} a_{1} & \cdots& a_{9} \end{matrix} \right]^{T}$ and $\boldsymbol{b}=\left[ \begin{matrix} b_{1} & \cdots& b_{9} \end{matrix} \right]^{T}$ are controlled by the settings of the phase shifters within the MZIs.

In operation as a self-configuring processor here, we presume first that we shine in some light beam that results in some vector ***u*_1_** of complex amplitudes in the input waveguides. We illustrate such a processor in Fig. S1, here for the smaller case of five input waveguides for simplicity, but the concept can be extended to an arbitrary number of input waveguides. In using the processor, we proceed first by a “self-configuration” process to set the MZIs in the first diagonal row. Explicitly, starting from the bottom MZI in the first diagonal row, MZI11, we adjust the phase shifters in that MZI so that no power emerges from the lower “drop port” output P11. If the beamsplitters in the MZI are 50:50, such an adjustment is always possible for any specific pair of input amplitudes for that MZI, and it can be achieved by two successive power minimizations in that lower output, one by adjusting the input phase shifter on the input waveguides, and the second by adjusting the internal phase shifter on one of the MZI internal arms; this second minimization will take the power at P11 to zero. As a result of this first step, all the power in the lowest two input guides is now present on the upper output waveguide of MZI11. We then similarly configure MZI12 to minimize the power to zero at P12, thus combining all the power from the lower three waveguides to the upper output of MZI12. We can then proceed similarly up the remaining MZIs in the diagonal line, with the result that all the power in the 5 input waveguides now emerges from the top output waveguide; this operation can be described as a “self-aligning beam coupler” [2].


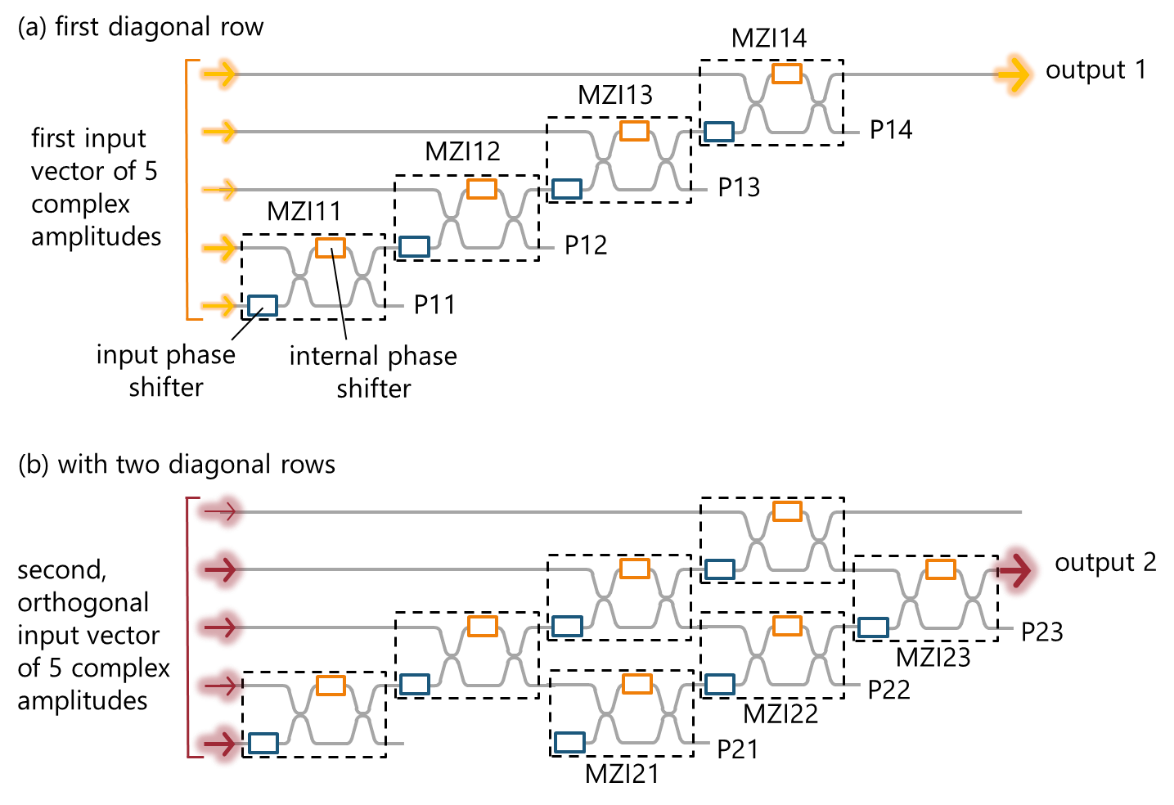


Figure S1. Operating principle of the processor, illustrated for the smaller case of 5 input waveguides. (a) The first diagonal line of MZIs, MZI11 to MZI14, with “drop-port” outputs P11 to P14. (b) The processor with two diagonal lines of MZIs, now adding MZI21 to MZI23.

A second important behavior of this processor is what happens if, while holding these settings of the first diagonal row of MZIs, we shine a second beam into this device, and specifically a second beam for which the vector of amplitudes ***u*_2_** in this second beam is orthogonal to the first beam ***u*_1_** – that is, for which the product $\boldsymbol{u}_{\boldsymbol{2}}^{\dagger}\boldsymbol{u}_{\mathbf{1}}=0$ (where the dagger “†” here represents the Hermitian adjoint or “conjugate transpose” of the vector). In this case, to the extent that this processor can be treated as lossless, *none* of the power in this second input beam can appear in the “top” output waveguide. In fact, if any such power did appear in the top output guide, it would violate the second law of thermodynamics, combining two (possibly mutually incoherent) beams losslessly into one, which would allow us to heat up a hotter body with two cooler ones; also, it would lead to non-uniqueness if we were to run this system backwards – which vector should we get out on the left if we shine light backwards into the top waveguide? Hence, *all* the power in the second beam ***u*_2_** must appear in the “lower” outputs, P11 to P14, of the MZIs in the first row. However, those outputs are connected to a second row of MZIs, MZI21 to MZI23, and we can now simply self-align this second row to give all the power in the second output waveguide on the right by minimizing the powers (to zero) in outputs P21 to P23. Note, incidentally, that the first beam ***u*_1_** can remain present while this second diagonal row is being configured, and this process works regardless of whether the two beams ***u*_1_** and ***u*_2_** are mutually coherent. (We have shown them as being of different colors in Fig. S1, but this is for graphic clarity; the two beams can be at the same wavelength, though they do not have to be.)

Once we have done this, we can see from looking at the matrix multiplication *D* of eq. (S2) that we must have $\boldsymbol{a}=\alpha\boldsymbol{u}_{\boldsymbol{1}}$ and $\boldsymbol{b}=\beta\boldsymbol{u}_{\boldsymbol{2}}$, where *α* and *β* are constants that depend on the power of the two input beams; this is the mathematical requirement that leads to the separation of these two orthogonal vectors to the corresponding elements of the output matrix. The matrix *D* therefore consists of two orthogonal rows that are simply proportional to the Hermitian adjoints of the orthogonal vectors that have been separated. To the extent that this processor is lossless, these vectors ***a*** and ***b*** are normalized (i.e., of unit “length”), and the matrix *D* is unitary. Generally, all the orthogonality and self-configuring properties are conserved if the loss is equal on all paths through the system, so in practice *D* can be unitary “within a factor” and the system still works. It is, of course, possible to calculate all the settings of the phase shifters in the MZIs that will give the necessary elements in *D*, and the algebra for that is straightforward (see, e.g., [3], [4]), but we never have to perform these calculations when we set this system up by self-configuration as we do here. The matrix *D* is a consequence of the orthogonal vectors we chose to separate; it is set up effectively by this physical self-configuration process, and it not otherwise required in the operation of the device, even though it gives a mathematical way to look at the resulting process.

In the implementation here, we make use of mostly-transparent detectors (see Fig. 1e of the main text) [5] at the various “drop ports” – so ports P11 to P14 and P21 to P23 – in the power minimization. So, these detectors can remain in place even as we minimize successive diagonal lines of MZIs. It is also possible to run these self-configuring algorithms based on maximizing the power at the outputs [2] rather than minimizing power at these internal detectors on the “drop ports”.

S2 Orthogonality conditions for the direction-diversity receiver

The limit to the capability of separating two overlapped beams is related to the orthogonality of the complex vectors of amplitudes ***u*_1_** and ***u*_2_** that are sampled by the 2D array feeding the photonic processor. If two different beams lead to orthogonal vectors, that is if $\boldsymbol{u}_{\boldsymbol{2}}^{\dagger}\boldsymbol{u}_{\boldsymbol{1}}=0$, the two beams can be separated with no mutual crosstalk, otherwise some crosstalk will be observed at the output ports of the processor.

Fig. S2. Schematic view of the phase front sampled by a 2D antenna array in the case of two beams arriving from two different directions.

In the case of the *direction-diversity receiver*, where two identically shaped (Gaussian) beams, sharing the same wavelength and the same polarization, arrive overlapped onto the 2D antenna array from different directions, orthogonality is uniquely given by the tilted phase front of the two beams at the plane of the 2D array. To explain the concept, whose theoretical aspects are well known in the field of phased array antennas, let’s consider the schematic of Fig. S2. Without loss of generality the direction of arrival of the first “reference” beam (TX1) is assumed aligned to the *x* axis of the 2D array, while a second beam arrives from a direction identified by an angle $\theta$ with respect to the first beam. A relative phase delay $\delta$ is observed between the phases of the complex fields sampled by adjacent antennas at different *y* coordinates on the array plane. For the reference beam ***u*_1_** such phase delay is zero ($\delta_{1}=0$), while for the second beam ***u*_2_** the phase delay is $\delta_{2}=kL\sin\theta,$ where $k = 2\pi/\lambda$ is the free-space wavevector and *L* is the spacing between the antennas along the *y* direction. If the direction of arrival $\theta$ is such that $\delta_{2}=2m\pi$ (*m* = 1, 2, …) the two vectors of sampled amplitude ***u*_1_** and ***u*_2_** are indistinguishable (in this case the scalar product $\boldsymbol{u}_{\boldsymbol{2}}^{\dagger}\mathbf{u}_{\mathbf{1}}$is maximum). For the array considered in this work, where *L* = 35 μm (corresponding to $22.6\lambda$, where λ is the free space wavelength), this condition is achieved when

$\sin\theta\approx\theta=\frac{\delta_{2}}{kL}=\frac{2m\pi}{kL}=\frac{2m\pi}{\frac{2\pi}{\lambda} 22.6\lambda}=0.044 m [rad]$ (S3)

corresponding to a set of angles that are multiples of 2.5°. These angles provide the direction of the diffraction orders (grating lobes) along the *x* and *y* directions, as shown in the simulations of Fig. S3. Therefore, if the two beams are overlapping in the diffraction orders, the photonic processor would not be able to distinguish them, while being orthogonal in their inner product over the continuous case.

Orthogonality conditions for the sampled beams ***u*_1_** and ***u*_2_** can be simply derived in the case of beams with a constant amplitude across the array elements. From the theory of uniform phased arrays, orthogonality along a given direction (for instance *y*) is achieved when $\delta=kL\sin\theta_{m}$ = $\frac{2m\pi}{M_{y}}$ (with *m* = 1,2, …, $M_{y}$-1), where $M_{y}$ is the maximum number of elements in that direction (the same condition applies in the orthogonal *x* direction). In our case $M_{y}$ = 3 and this condition leads to

$\sin\theta_{m} \approx\theta_{m}=\frac{2m\pi}{M_{y}kL}=\frac{2m\pi}{3\frac{2\pi}{\lambda} 22.6\lambda}=0.0145 m [rad]$. (S4)

resulting in $\theta_{1}$ = 0.0145 [rad ] (0.83°) for *m* = 1 and $\theta_{2}$ = 0.029 [rad] (1.66°) for *m* = 2.

Since the photonic processor can control both the amplitude and the phase of the field coupled by each optical antenna, the amplitude excitation profile of the array can be exploited as a further degree of freedom. Figure S3 shows the simulated far-field pattern of the 2D optical antenna array employed in this work when the amplitude of the field radiated by the grating couplers is distributed uniformly (a) or non-uniformly and according to a Gaussian profile (d). The values of the field amplitudes (normalized to that of the central antenna) are indicated in the figure, while all the phases are zeros (thus, the main lobe is located in the center of the far-field pattern). Results show that in case of uniform excitation, the normalized radiated power in the far field at 1.25° azimuth (or elevation) angle is about 20 dB below the main lobe [panels (b) and (c)], which is due to the presence of a side-lobe in this angular position of the far-field pattern. The angles in Eq. (S4) provide the angular positions of the zeros of the far-field radiation pattern along the *x* and *y* directions. In case of non-uniform excitation [panels (e) and (f)], it can be seen that the side lobe can be further reduced ($<30 \text{dB}$) and replaced by a large zero of radiation. Due to reciprocity, this behaviour is maintained when the antenna array is used at the receiver side, as in the direction-diversity receiver. Therefore, the high optical crosstalk suppression (> 25 dB) observed in the experiments of Fig. 2 of the main text is achieved because the mesh can control both the amplitude and phase of the field coupled by each grating coupler of the 2D optical antenna array.

Figure S3. Simulation of the far-field radiation pattern of the 2D optical phase array in case of uniform (a)-(c) and non-uniform (d)-(f) amplitude excitation of the array. Curves in panels (c) and (f) refer to the 1D far-field profile along the azimuth angle (blue dashed line) and the elevation angle (solid red line), respectively.

S3. Experimental setup

In this section the experimental setups employed for the experiments reported in the main text are described in detail.

**S3.1 Direction-diversity receiver**

Figure S4 shows a schematic of the experimental setup used to test the direction-diversity receiver. Two free-space optical beams are transmitted by two identical fiber coupled collimators (TX1 and TX2) that generate two Gaussian beams with a waist of 1.12 mm. These two beams are imaged to the two ports of a 50/50 beam splitter (BS1) via a 4f lens system consisting of a bi-convex lens with a focal length *f*_3_ = 250 mm. Deliberately, the two beams are not overlapping in the beam splitter plane (the mutual spacing being 2.1 mm), though they arrive overlapped at mesh inputs from different directions with a relative angle of 1.25°. The lens system between BS1 and the photonic chip is used to match the shape and size of the Gaussian beams in center of BS1 to the collimated far field of the 2D optical antenna array. The system includes a pair of bi-convex lenses (*f*_1_ = 50 mm) that are used in Fourier transforming condition to create the collimated far field of the 2D optical antenna array in the plane *P*_1_, at the distance of 10 cm from the photonic chip, and the image plane of the 2D array at plane *P*_2_ (20 cm away). Another bi-convex lens (*f*_2_ = 75 mm) creates the required matching condition on BS1 at a distance of 350 mm from the chip. Therefore, direction diversity implies that in the “far-field” planes (*P*_1_ and *P*_3_) the two Gaussian beams are not spatially overlapped; however, in the 2D array plane on the chip surface (as well as in the image plane *P*_2_) they are spatially overlapped.

The 2D far-field profile shown in Fig. 2(c) of the main text is acquired using a near-infrared (NIR) camera focused on the plane P1 after a 92/8 beam splitter (BS2). In this case the light beams are shone backward, injecting the light from the single-mode waveguide ports WG_in_ of the programmable mesh, as indicated by the dashed arrows.

Figure S4. Experimental setup employed for the demonstration of the direction-diversity receiver.

**S3.2 Mode-diversity receiver**

In order to generate the two spatially overlapped orthogonal beams employed to test the mode-diversity receiver, the setup of Fig. S4 is slightly modified as shown in Fig. S5.

A phase mask made by etching a 500 $\mu m$ thick silica substrate is positioned in front of the fiber collimator TX2 to introduce a 0-π phase jump on the vertical axis of the beam. Etch depth of 1.663 $\mu m$ (on one side) corresponds to π phase shift when compared with the unetched surface of the substrate (reference phase or 0 phase shift). As a result, the Gaussian beam at the output of the fiber collimator (HG_00_) is transformed to a higher-order HG_10_-like mode (Mode 2). Actually, it is not exactly a HG_10_ mode because the amplitude pattern does not have exactly that form; rather, a sudden π-phase shift is introduced between one side of the beam and the other on the vertical axis of the beam, giving a “two-bumped” beam with the same anti-symmetry and phase behavior as a HG_10_ mode.

The second difference in the setup is that the positions of the two fiber collimators are optimized in such a way that the two modes (Mode 1 and Mode 2) are spatially overlapped in the far-field planes (P1 and P3) and arrive on the 2D antenna array with the same direction.

Figure S5. Experimental setup employed for the demonstration of the mode-diversity receiver.

**S3.3 Mode-mixed receiver**

The setup employed to test the mode-mixed receiver is shown in Fig. S6. Referring to the experimental setup of S5, in the far-field plane P1 the fundamental mode HG_00_ (Mode 1) and the higher-order HG_10_-like mode (Mode 2) are spatially overlapped. If the phase mask in front of collimator TX2 is rotated -45°, the axis of the Mode 2 in P1 will be rotated accordingly. Now, we introduce in the plane P1 (instead of the beam splitter BS2) an additional 0-π phase mask which is rotated by 45° with respect to the vertical axis. After passing through this mask, the fundamental mode HG_00_ is converted to a 45° rotated HG_10_-like mode (Mode 3), while the higher-order HG_10_-like mode (-45° rotated) is transformed to a 45° rotated HG_11_-like mode (Mode 4). In other words, the second phase mask performs a linear transformation between pairs of orthogonal modes, that is a mode conversion.

Figure S6. Experimental setup employed for the demonstration of the mode-mixed receiver.

S4. Geometrical loss of the 2D array

A major contribution to the overall loss of the free-space setup is given by the fill factor of the array (geometrical loss), that is given by the ratio between the overall aperture of the array (M times the aperture of the single grating coupler) and the size of the optical beam impinging onto the array.

To evaluate the geometrical loss, we simulated the beam evolution through the entire optical setup. In Fig. S7 we show the OpticStudio (Zemax) model of one of the branches of the optical setup of Fig. S4, from a fiber collimator (on the left) to the plane of the 2D array of the photonic chip (on the right). According to this scheme, the first lens on the left is in a $4f_{3}$ configuration with $f_{3}=250\text{mm}$; the other three lenses are in a Fourier transforming configuration with $f_{2}=75\text{mm and }f_{1}=50\text{mm}$, respectively. The ray traces are shown in the layout considering an aperture for the light source with the same divergence as the initial Gaussian beam of the fiber collimator (TX1 or TX2).


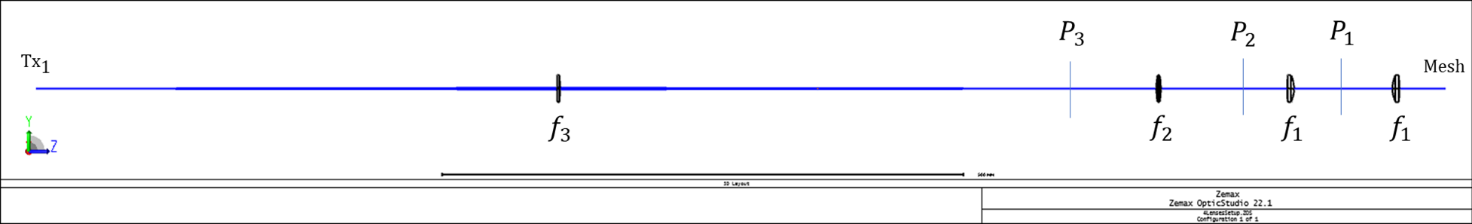


Fig S7. Layout of the simulated optical setup in OpticStudio (Zemax)

The initial beam has a beam diameter of $1.12 \text{mm}$ with full divergence of $0.1^{\circ}$, as provided by the fiber collimator. The beam profile at the receiver plane, which is placed at the back focal plane of the last lens (on the right), can be seen in Fig. S8. The beam size, defined here as the beam radius at the $1/{e^{2}}$ intensity, is $214 \text{m}\text{m}$. We have also calculated the fiber coupling efficiency (overlap integral) approximating the near field of a grating coupler as a Gaussian beam with a waist of $10 \text{m}\text{m}$. The coupling efficiency is $-20.6 \text{dB}$ for a single grating coupler placed at the center of the received beam and $-21.6 \text{dB}$ for the ones shifted by 70 μm on the horizontal or vertical line, and $-21.2 \text{dB}$ for the ones shifted by 50 μm on the 45° diagonal. Therefore, the total geometrical loss of all 9 grating couplers is about $11.8 \text{dB}$. Due to reciprocity, this value gives the power that is “lost” in the grating lobes of the far-field pattern, when reversing the propagation direction from the photonic processor to the fiber collimator.


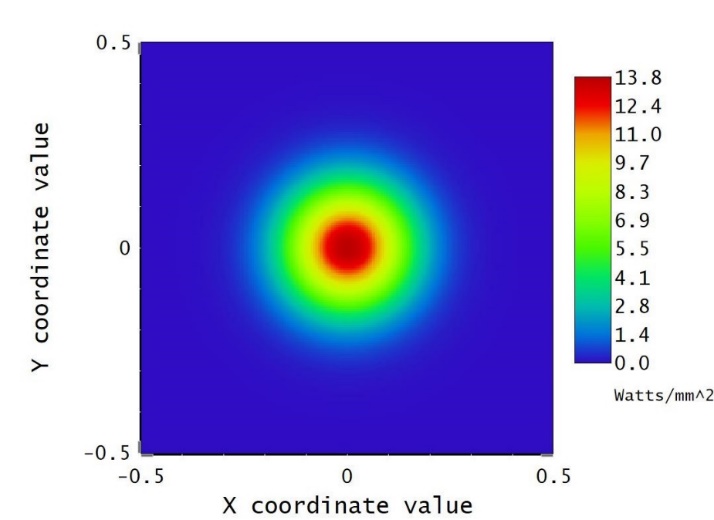


Fig. S8. Received beam profile at the receiver plane of the antenna array­­

S5 Wavelength dependence of the photonic processor

The MZIs of the photonic processor are designed to operate as balanced tunable couplers and their spectral response is mainly limited by the wavelength dependence of the directional couplers. In Fig. S9, we show the wavelength dependence of a single MZI in bar and cross states. We observe a 3-dB bandwidth of more than 60 nm in 100% transmission (bar port) and about 22 nm of bandwidth at -20 dB transmission (cross port). The electrical power consumption to thermally switch the MZI tuneable coupler, that is to provide a π-shift by the thermal actuator, is 22 mW.

Fig. S9. Wavelength dependence of a balanced MZI of the photonic processor.

Figure S10 shows the spectral response of the entire photonic processor when it is configured to separate two beams in a wavelength range centered at 1550 nm. The wavelength dependence of the transmission of the extracted Mode 1 (blue circles) and of the rejected Mode 2 (blue squares) at output port WG1 follows the spectral behaviour of the elementary MZI tuneable coupler. The bandwidth is further narrowed by some geometrical unbalances in the length of the waveguides connecting the 2D optical array to the processor which translates to a more pronounced wavelength dependence. However, if the wavelength of the incoming beams is changed in the 1535 nm – 1570 nm range, the working point of the processor can be adaptively adjusted by retuning the phase shifters in order to optimize mode separation at the working wavelength, as shown by the red curves in Fig. S10.

The wavelength dependence of the photonic processor can be reduced by using wide bandwidth directional couplers or using MMIs instead of directional couplers in each MZI.

Fig. S10. Wavelength dependence of the photonic processor when it is tuned to extract Mode 1 (blue circles) at output port WG1 at a wavelength of 1550 nm (blue squares indicate transmission of Mode 2 to the same output port WG1). Red curves show the maximum transmission (Mode1, circles) and maximum rejection (Mode 2, circles) at port WG1 when the processor is reconfigured at the considered wavelength.

Another limitation to the spectral range of the device is given by the bandwidth of the grating coupler, which in our work has a 3 dB bandwidth of 40 nm. The free-space optical components employed in the experimental setup (lenses, mirror, and beam splitters) are almost wavelength independent in the whole wavelength range considered (1535 nm – 1570 nm).

**References**

| [1] A. Michaels and E. Yablonovitch, "Inverse design of near unity efficiency perfectly vertical grating couplers," *Opt. Express,* vol. 26, 4766-4779, 2018.  [2] D. A. B. Miller, “Self-aligning universal beam coupler,” *Opt. Express ,* vol. 21, pp. 6360-6370, 2013. |
| --- |
| [3] D. A. B. Miller, “Self-configuring universal linear optical component,” *Photon. Res.,* vol. 1, pp. 1-15, 2013.  [4] D. A. B. Miller, “Analyzing and generating multimode optical fields using self-configuring networks,” *Optica,* vol. 7, pp. 794-801, 2020.  [5] F. Morichetti, S. Grillanda, M. Carminati, G. Ferrari, M. Sampietro, M. J. Strain, M. Sorel and A. Melloni, "Non-invasive on-chip light observation by contactless waveguide conductivity monitoring," *IEEE Journal of Selected Topics in Quantum Electronics,* vol. 20, no. 4, pp. 292-301, 2014. |
